# Inhomogeneous phases in coupled electron-hole bilayer graphene sheets: Charge Density Waves and Coupled Wigner Crystals

**DOI:** 10.1038/s41598-017-11910-w

**Published:** 2017-09-14

**Authors:** M. Zarenia, D. Neilson, F. M. Peeters

**Affiliations:** 1 Department of Physics, University of Antwerp, Groenenborgerlaan 171, B-2020 Antwerpen, Belgium; 2 Dipartimenti di Fisica e di Farmacia, Università di Camerino, 62032 Camerino, Italy

## Abstract

Recently proposed accurate correlation energies are used to determine the phase diagram of strongly coupled electron-hole graphene bilayers. The control parameters of the phase diagram are the charge carrier density and the insulating barrier thickness separating the bilayers. In addition to the electron-hole superfluid phase we find two new inhomogeneous ground states, a one dimensional charge density wave phase and a coupled electron-hole Wigner crystal. The elementary crystal structure of bilayer graphene plays no role in generating these new quantum phases, which are completely determined by the electrons and holes interacting through the Coulomb interaction. The experimental parameters for the new phases lie within attainable ranges and therefore coupled electron-hole bilayer graphene presents itself as an experimental system where novel emergent many-body phases can be realized.

## Introduction

The interplay between superconducting and charge density wave (CDW) phases that is often observed in connection with High-Temperature superconductors, is attracting considerable attention. It has been argued that details of the very elaborate crystal structures typical of High-Temperature superconductors play a central role in determining the properties of the CDWs. A polarizable background is needed to drive the CDW phase, and the crystal lattice provides this background. However the complexity of the crystals in these materials, makes it challenging to identify the competing contributions^1–5^. We report in this manuscript on a, by far, simpler system exhibiting the same association of superfluid and CDW phases, but a system in which the polarizable background is uniform, so there is no intricate background structure that could determine the properties of the CDW.

There exist several studies pertinent to inhomogeneous phases in coupled two-dimensional-electron-gas (2DEG) layers^6–8^. Despite these early attempts, however, there are no studies of the CDW phase for small electron-hole layer separations where interlayer correlation effects will be strong. The earliest approaches could not treat strong interlayer coupling^7, 8^, while for QMC calculations, it is impractical to study an inhomogeneous ground state like a CDW with oscillations of both unknown period and amplitude^6^.

Here we propose two strongly coupled two-dimensional (2D) bilayers of graphene (BLG), one bilayer containing electrons and the other holes, as an experimentally accessible system to observe the CDW phase. An electron and hole bilayer graphene has a quadratic dispersion around the Fermi level *E*_*F*_ for densities *ρ* < 4 × 10^12^ cm^−2^ ^9^. The electron and hole effective masses are matched, lying in the range $0.03\mathop{ < }\limits_{ \tilde {}}{m}^{\ast }/{m}_{e}\mathop{ < }\limits_{ \tilde {}}0.05$ ^10, 11^. A lower limit of experimental attainable densities is $\rho \mathop{ > }\limits_{ \tilde {}}{10}^{10}$ cm^−2^ ^12–14^. The electron and hole densities are controlled by top and back metal gates. The electric field from the metal gates opens up an energy band gap between the parabolic conduction and valence bands^15^. We consider bilayer graphene embedded in a hexagonal Boron Nitride (h-BN) dielectric which is important to ensure high charge mobility and a large potential barrier between the graphene bilayers. The valley degeneracy is *g*_*v*_ = 2 and the dielectric constant *κ* = 3^16^. The separation barrier between the bilayer sheets can be as little as *d* = 1 nm with no significant leakage from tunneling^17^. Such separations are much less than the effective Bohr radius, ${a}_{B}^{\ast }\sim 11$  nm for bilayer graphene. At the densities of interest to us, the average spacing between the carriers in each bilayer is much greater than the lattice constant of graphene, making details of the graphene lattice structure unimportant. We will consider only equal electron and hole densities. Furthermore, high quality graphene lattices with extremely low disorder can be fabricated^18, 19^.

In this paper we find, besides the superfluid phase discussed in refs 20–23, that there are two new inhomogeneous ground-state phases: (i) a strongly-coupled one-dimensional charge density wave (1D-CDW), and (ii) a coupled electron-hole Wigner crystal (c-WC). Because of the simple structure of the system, the drivers for all these phases are very simple. First, there is the Coulomb attraction between electrons and holes from the two bilayers which is controlled by the layer separation *d*. Second, there is the Coulomb repulsion between pairs of electrons or pairs of holes within each bilayer which is controlled by the carrier density *ρ* in the bilayer.

**Figure 1:**
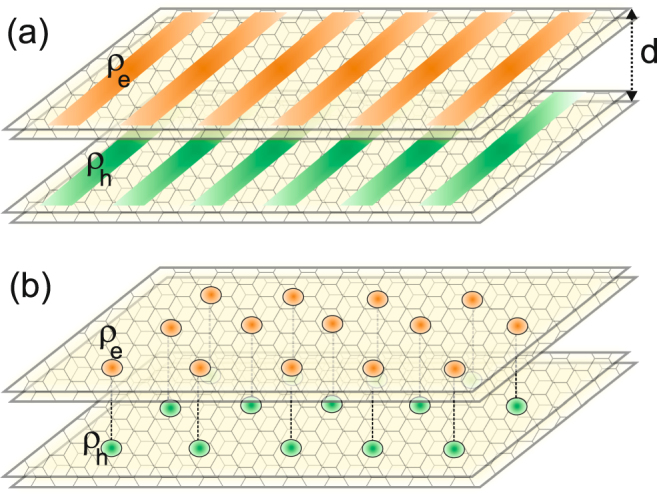
Schematic illustration of electron and hole density distribution in (**a**) charge density wave phase and (**b**) coupled Wigner crystal.

We find that the 1D-CDW, with density modulations in one planar direction, is always more stable than the two-dimensional CDW. (We also find that in regions of phase space where the liquid phase is more stable than the 1D-CDW, that the liquid phase has a lower energy than the two-dimensional CDW). As would be expected, for the 1D-CDW phase, the planar density modulation in the two bilayers share the same periodicity and they are in phase (see Fig. 1(a)), since this ensures a maximum attractive potential energy gain from the electron-hole interactions. In contrast with the case of High-Temperature superconductors, the holes from one bilayer act as a perfectly symmetric polarizable background for the electrons in the other bilayer, and *vice versa*. This property makes our CDW phase uniquely different from CDW phases in other systems.

In the c-WC phase, an electron Wigner crystal in one bilayer couples to a hole Wigner crystal in the other bilayer. The electron and hole sites of the c-WC will align, again to maximize the attractive potential energy gain from the electron-hole interactions, so each lattice site in the hole Wigner crystal lies directly opposite a lattice site in the electron Wigner crystal (see Fig. 1(b)).

When the separation *d* between bilayers is not too large, the Coulomb attraction between the electrons and holes will generate strongly bound electron-hole pairs that can condense into a quantum coherent superfluid state^23, 24^. However, for densities above a cut-off density, strong screening is predicted to suppress the superfluidity^23, 25^.

As the layer separation *d* is increased, an interesting phase transition should occur from the superfluid phase to the 1D-CDW phase. While CDWs are conventionally treated as a classical phase, Bardeen^26^ argued for extending the coherent quantum state interpretation from superconductors to include 1D-CDWs. Latyshev *et al*.^27^ subsequently reported observation of Aharonov-Bohm-like oscillations in the original CDW material NbSe_3_^28^.

At still lower densities, the transition with increasing *d* would, instead, be from the superfluid to the c-WC phase. This transition would be driven by the competition between the repulsive interactions between like-charge carriers in one bilayer, which favour the Wigner crystal, and the attractive interactions between electrons and holes, which favour pair formation and superfluidity. At very small densities *ρ* and for small *d*, the electron-hole pairs are compact and nearly neutral. The residual repulsive interactions within a bilayer are through weak dipolar interactions, so a superfluid phase would be favoured. However at larger *d* for the same *ρ*, the attractive electron-hole interactions between layers become weak compared with the repulsive interactions between like-carriers within each bilayer. The repulsive interactions favour the WC phase, and the system would make a transition from the superfluid to the quantum c-WC phase. At extremely low densities, the Wigner crystal would become classical, and at that point there would be an interesting transition from the quantum coherent superfluid condensate to a classical Wigner crystal phase.

At sufficiently large *d*, the bilayers will decouple into two independent bilayers, so for decreasing *ρ*, the Fermi liquid phase will make a transition directly to a Wigner crystal that is only very weakly coupled.

## Methods

It is impractical to investigate the CDW phase in this system using quantum Monte Carlo (QMC) techniques, since the amplitude, periodicity and dimensionality, are not *a priori* known. In a recent work^29^, a new and fast interpolation scheme was introduced to obtain accurate correlation energies for the one-layer 2DEG. The method gives excellent agreement with QMC correlation energies for both one-valley and two-valley two-dimensional-electron-gas systems.

In this approach the correlation energy *E*_c_[*ρ*] is determined by an interpolation of *W*_*α*_[*ρ*], the potential energy functional of *ρ* without the Hartree contribution, of a fictitious system that interacts with a Coulomb interaction scaled by a coupling constant factor *α*. The interpolation is between ${W}_{{\mathrm{lim}}_{\alpha \mathrm{=0}}}[\rho ]$ (weakly-interacting) and ${W}_{{\mathrm{lim}}_{\alpha =\infty }}[\rho ]$ (strongly-interacting)^30^,

1 $${E}_{{\rm{c}}}[\rho ]=({W}_{0}-{W}_{\infty })\,[\frac{\sqrt{1+2X}-1}{X}-1],$$

where $$X={{\rm{d}}{W}_{\alpha }/{\rm{d}}\alpha |}_{\alpha =0}/({W}_{\infty }-{W}_{0})$$.

Here, we extend the approach from ref. 29 (see Sec. S1 of Supplementary for a brief discussion of the approach) to coupled electron-hole bilayer graphene sheets. For the two bilayers, the exact Random Phase Approximation for the above expressions in the weakly-interacting limit, *α* → 0, are given by

2 $${W}_{0}[\rho ]=\sum _{{\boldsymbol{q}}}\,{v}_{q}\,[-\frac{\hslash }{2\pi \rho }{\int }_{0}^{\infty }\,{\rm{d}}\omega \,\sum _{\ell ,\ell ^{\prime} }\,{\chi }_{0}^{\ell \ell ^{\prime} }({\boldsymbol{q}},i\omega )-1]$$

3 $${\frac{{\rm{d}}{W}_{\alpha }}{{\rm{d}}\alpha }|}_{\alpha =0}=-\frac{\hslash }{2\pi \rho }\,\sum _{{\boldsymbol{q}}}\,{v}_{q}\,{\int }_{0}^{\infty }\,{\rm{d}}\omega \,\sum _{\ell ,\ell ^{\prime} }\,\frac{{\rm{d}}{\chi }_{\alpha }^{\ell \ell ^{\prime} }({\boldsymbol{q}},i\omega )}{{\rm{d}}\alpha }+{W}_{0}^{^{\prime} \mathrm{(2)}}$$

with

4 $$\tfrac{1}{{\chi }_{\alpha }^{\ell \ell ^{\prime} }({\boldsymbol{q}},i\omega )}=\tfrac{1}{{\chi }_{\alpha }^{\ell }({\boldsymbol{q}},i\omega )}{\delta }_{\ell \ell ^{\prime} }-\alpha {v}_{\ell \ell ^{\prime} }(q)\,\mathrm{(1}-{\delta }_{\ell \ell ^{\prime} }),\,{\chi }_{\alpha }^{\ell }({\boldsymbol{q}},i\omega )=\tfrac{{\chi }_{0}^{\ell }({\boldsymbol{q}},i\omega )}{1-\alpha {v}_{q}{\chi }_{0}^{\ell }({\boldsymbol{q}},i\omega )},$$

where *v*_11_ = *v*_22_ = *v*_*q*_, *v*_12_ = *v*_21_ = −*v*_*q*_ *e* ^−*qd*^, with *v*_*q*_ = *e* ^2^/*κq*. The term ${W}_{0}^{^{\prime} \mathrm{(2)}}$ in Eq. (3) is determined from the contribution of the second-order correction to the correlation energy ${E}_{{\rm{c}}}^{\mathrm{(2)}}$. It is given by ${W}_{0}^{^{\prime} \mathrm{(2)}}=2{E}_{{\rm{c}}}^{\mathrm{(2)}}$ ^29^, with ${E}_{{\rm{c}}}^{\mathrm{(2)}}\approx {g}_{v}\times 0.105$ Hartree, independent of the layer separation. ${\chi }_{0}^{\ell }({\boldsymbol{q}},i\omega )={\chi }_{0}({\boldsymbol{q}},i\omega )$ is the non-interacting density-density response function for layer $\ell$ and is given by,

5 $${\chi }_{0}({\boldsymbol{q}},i\omega )={g}_{s}{g}_{v}\,\sum _{s,s^{\prime} }\,\int \,{{\rm{d}}}^{2}{\boldsymbol{k}}\frac{{f}_{k}-{f}_{{\boldsymbol{k}}+{\boldsymbol{q}}}}{{\varepsilon }_{{\boldsymbol{k}}}^{s}-{\varepsilon }_{{\boldsymbol{k}}+{\boldsymbol{q}}}^{s^{\prime} }+i\hslash \omega +i\eta }{F}_{{\boldsymbol{k}},{\boldsymbol{k}}+{\boldsymbol{q}}}^{s,s^{\prime} }.$$

*f*_***k***_ is the Fermi-Dirac function for the wave vector ***k***, *g*_*s*_ and *g*_*v*_ are the spin and valley degeneracies, *η* > 0 is an infinitesimal number, and ${F}_{{\boldsymbol{k}},{\boldsymbol{k}}^{\prime} }^{s,s^{\prime} }=(1+ss^{\prime} \,\cos \,2\varphi )/2$ is the wavefunction overlap factor in BLG with *ϕ* the angle between *k* and *k*′. ${\varepsilon }_{{\boldsymbol{k}}}^{s(s^{\prime} )}$ are the electron (*s* = +1) and hole (*s* = −1) energy bands in BLG defined as,

6 $${\varepsilon }_{{\boldsymbol{k}}}^{\pm }={U}_{1}\mathrm{/2}+{U}_{2}\mathrm{/2}\pm \sqrt{{({U}_{1}-{U}_{2})}^{2}\mathrm{/2}+{(\hslash {v}_{F}k)}^{4}/{\gamma }^{2}}.$$

which are the eigenenergies of the 2 × 2 Hamiltonian,

7 $$H=-\frac{{(\hslash {v}_{F})}^{2}}{\gamma }\,[\begin{array}{cc}0 & {({k}_{x}+i{k}_{y})}^{2}\\ {({k}_{x}-i{k}_{y})}^{2} & 0\end{array}]+[\begin{array}{cc}{U}_{1} & 0\\ 0 & {U}_{2}\end{array}].$$

This can properly describe BLG at low energies^9, 31^. *γ* ≈ 400 meV is the dominant interlayer coupling between the sublattices *A* and *B*′ from the upper and lower layers, and *v*_*F*_ = 10^6^ m/s is the Fermi velocity. *U*_1_ and *U*_2_ are the electrostatic potentials applied to the upper and lower layers which are required to induce electrons (or holes) in each BLG.

We note that the transition to the inhomogeneous phases occurs at very low densities, corresponding to low energies, e.g. for $n\mathop{ < }\limits_{ \tilde {}}1.5\times {10}^{11}$ cm^−2^, ${E}_{F}\mathop{ < }\limits_{ \tilde {}}5$ meV. We show in the Supplementary Information that for such low energies, whenever there is a significant gap in the BLG spectrum, ${\rm{\Delta }}U=|{U}_{1}-{U}_{2}|\gg {E}_{F}$, *χ*_0_ can be evaluated by considering only the conduction band *E*_+_. For this reason we neglect the influence of the hole band in the calculation of Eq. (5), considering only the conduction band in the BLG with quadratic dispersion.

In the limit of strong interactions, *α* → ∞, the ground state is the classical WC. We can treat the classical crystal as a collection of neutral unit cells, each cell with an electron or hole at its centre and surrounded by a charged disk of uniform neutralizing background of radius ${r}_{0}=1/\sqrt{\pi \rho }$. Then *W*_∞_ is obtained from a straightforward electrostatic calculation,

8 $${W}_{\infty }={E}_{e\oplus }+{E}_{h\ominus }+{E}_{\oplus \oplus }+{E}_{\ominus \ominus }+{E}_{e\ominus }+{E}_{h\oplus }+{E}_{eh}+{E}_{\ominus \oplus },$$

where

9 $$\begin{array}{rcl}{E}_{e\oplus } & = & -\frac{2}{{r}_{s}}\equiv {E}_{h\ominus }\\ {E}_{\oplus \oplus } & = & \frac{8}{3\pi {r}_{s}}\equiv {E}_{\ominus \ominus }\\ {E}_{e\ominus } & = & \frac{2}{{r}_{s}^{2}}[\sqrt{{d}^{2}/{a}_{B}^{\ast 2}+{r}_{s}^{2}}-d/{a}_{B}^{\ast }]\equiv {E}_{h\oplus }\\ {E}_{eh} & = & -\frac{{a}_{B}^{\ast }}{d}\\ {E}_{\ominus \oplus } & = & -\frac{1}{{\pi }^{2}{r}_{s}^{4}{a}_{B}^{\ast 3}}\,\int \,{d}^{2}r\,\int \,\frac{{d}^{2}r^{\prime} }{\sqrt{{|{\boldsymbol{r}}-{\boldsymbol{r}}^{\prime} |}^{2}+{d}^{2}}}.\end{array}$$

We use Hartree units throughout the paper and we introduce the parameter ${r}_{s}={r}_{0}/{a}_{B}^{\ast }$.

Equations (2), (3) and (8), when inserted in Eq. (1), give us the correlation energy *E*_c_[*ρ*]. To obtain the total ground state energy *E*[*ρ*], we employ a Density Functional Theory formalism, for which

10 $$E[\rho ]=K[\rho ]+{E}_{{\rm{coul}}}[\rho ]+{E}_{{\rm{x}}}[\rho ]+{E}_{{\rm{c}}}[\rho ]+{E}_{{\rm{i}}},$$

where *K*[*ρ*], *E*_coul_[*ρ*], and *E*_x_[*ρ*] respectively denote the non-interacting kinetic energy, intra-layer Coulomb energy, and exchange energy functionals. The local-density approximation forms of these functionals are given by Eqs (10–13) in ref. 29, with valley index *g*_*v*_ = 2 for bilayer graphene. *E*_i_ is the inter-layer Coulomb interaction,

11 $${E}_{{\rm{i}}}[{\rho }_{1},{\rho }_{2}]=\frac{1}{2}\,\int \,{{\rm{d}}}^{2}r\,\int \,{{\rm{d}}}^{2}r^{\prime} \frac{[{\rho }_{1}({\boldsymbol{r}})-{\rho }_{0}]\,[{\rho }_{2}({\boldsymbol{r}}^{\prime} )-{\rho }_{0}]}{\sqrt{{|{\boldsymbol{r}}-{\boldsymbol{r}}^{\prime} |}^{2}+{d}^{2}}},$$

where ${\rho }_{\ell }({\boldsymbol{r}})$ is the charge density distribution in layer $\ell$.

We use Eq. (10) to obtain the ground state energy per particle $\varepsilon [\rho ]=E[\rho ]/\int \,{d}^{2}r{\rho }_{0}$ for the liquid phase with uniform density *ρ*(***r***) = *ρ*_0_, and for the non-uniform density distribution *ρ*(***r***) of the c-WC and the CDW phases.

For the c-WC phase, we take the variational form for the density distribution,

12 $$\rho ({\boldsymbol{r}})={\rho }_{0}\frac{{\beta }_{{\rm{WC}}}}{\pi }\,\sum _{m,n}\,\exp [-{\beta }_{{\rm{WC}}}{({\boldsymbol{r}}-m{{\boldsymbol{a}}}_{1}-n{{\boldsymbol{a}}}_{2})}^{2}],$$

where *m* and *n* are integers, and ***a***_1_ = *a*(1, 0) and ${{\boldsymbol{a}}}_{2}=a(-\mathrm{1/2,}\,\sqrt{3}\mathrm{/2})$ are the lattice vectors for the two-dimensional hexagonal lattice, with the lattice constant $a={a}_{{\rm{WC}}}=\sqrt{2/\sqrt{3}{\rho }_{0}}$ fixed by *ρ*_0_. This variational form is a superposition of Gaussians centred on the WC lattice sites. *β*_WC_ is our variational parameter that determines the degree of localization on each lattice site.

For the 2D-CDW we use the same variational form for *ρ*(***r***), Eq. (12), but now we take *a* as an additional variational parameter.

For the 1D-CDW phase, we take the variational form

13 $$\rho ({\boldsymbol{r}})={\rho }_{0}\frac{{\beta }_{{\rm{CDW}}}}{\pi }\,\sum _{m}\,\exp [-{\beta }_{{\rm{CDW}}}{({\boldsymbol{r}}-{\gamma }_{{\rm{CDW}}}\times m{{\boldsymbol{a}}}_{1})}^{2}],$$

with the amplitude *β*_CDW_ and the periodicity *γ*_CDW_ as the two variational parameters.

## Results and Discussions

**Figure 2:**
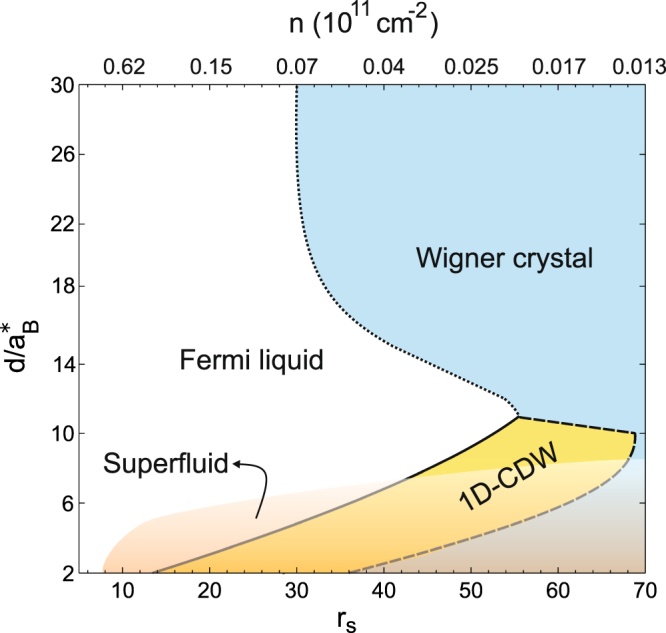
Zero temperature phase diagram for the ground state of the coupled electron-hole bilayer graphene system as a function of layer density parameter *r*_*s*_ and the separation between the layers *d*. The top x-axis gives the corresponding electron (hole) density. The transparent region at small *d*, schematically represents the superfluid phase (not calculated here) that would interplay with the 1D charge-density-wave (1D-CDW) and coupled Wigner crystal phases.

Figure 2 shows the phase diagram as a function of the inter-particle spacing *r*_*s*_ within each bilayer sheet and the separation *d* between the bilayer sheets. We see that for large $d/{a}_{B}^{\ast }\mathop{ > }\limits_{ \tilde {}}15$, the Fermi liquid is the ground state down to density *r*_*s*_ ≈ 32, where the transition to the c-WC occurs. This is as expected, since the coupling between the sheets is weak when $d/{a}_{B}^{\ast }\gg 1$, and consequently the results become similar to the results for a single isolated sheet^32, 33^.

When we decrease the separation *d* below $d/{a}_{B}^{\ast }=15$, the transition to the c-WC phase initially moves to lower density. This is caused by the increasing strength of the interlayer attractions when $d\mathop{ < }\limits_{ \tilde {}}{r}_{0}$. As a result the positions of the electrons and holes become more tightly correlated, and so there is increasing cancellation between their charges. This has the effect of reducing the electron-electron and hole-hole repulsion within each bilayer, which is what drives the transition to the WC. The long-range Coulomb repulsion between like-charges in the same bilayer is screened and tends towards the much weaker dipolar repulsion. The weaker repulsion makes it more difficult to form the c-WC, and the transition moves to lower density.

When *d* decreases below $d/{a}_{B}^{\ast }=12$, a new CDW phase interposes itself between the Fermi liquid and c-WC phases. With increasing *r*_*s*_ for fixed *d*, there are then two transitions: (i) from the Fermi liquid to the CDW phase, and (ii) from the CDW phase to the c-WC phase. As *d* is further decreased, both these transitions move to higher densities. The reason is that an increase in the strength of the interlayer electron-hole attraction favours formation of the inhomogeneous phases^7, 8^.

At sufficiently small *d*, the electron-hole attraction becomes strong enough for electron-hole bound pairs to form in significant numbers, and these should condense into a coherent superfluid state^6, 23^. It has been predicted that there is no BCS superfluid regime because for small ${r}_{s}\mathop{ < }\limits_{ \tilde {}}2.5$ there is very strong screening and this suppresses superfluidity^23^. As *r*_*s*_ increases above 2.5, the superfluid phase would first form directly in the BCS-BEC cross-over regime, and for larger *r*_*s*_ it would then evolve into the Bose-Einstein regime.

For $2.5\mathop{ < }\limits_{ \tilde {}}{r}_{s}\mathop{ < }\limits_{ \tilde {}}10$, the transition temperature for superfluidity at small *d*~1 nm can be large, i.e. >10 K^23^. However when ${r}_{s}\mathop{ > }\limits_{ \tilde {}}20$ the superfluid phase would occur only at extreme low temperatures, since the Kosterlitz-Thouless temperature *T*_*KT*_, which establishes an upper limit on the transition temperature for a 2D superfluid of only *T*_*KT*_ ≤ 40 mK by *r*_*s*_ = 20 even for the smallest *d*. We recall that *T*_*KT*_ decreases with *r*_*s*_ as ${r}_{s}^{-2}$. In practice, for such low transition temperatures, residual disorder in the graphene lattice is likely to kill any superfluidity. Furthermore, for *r*_*s*_ = 20, the predicted superfluid condensate fraction is already extremely small for all values of *d*. Even at $d/{a}_{B}^{\ast }=1$, the condensate fraction is only *c* = 0.02^6^, and *c* decreases even further for larger *d*. We conclude that for ${r}_{s}\mathop{ > }\limits_{ \tilde {}}20$, even if any superfluid should survive, it would most likely coexist in a phase separated state with the CDW or c-WC phase. In Fig. 2, a possible superfluid phase (not calculated here) has been schematically represented by the transparent area.

**Figure 3:**
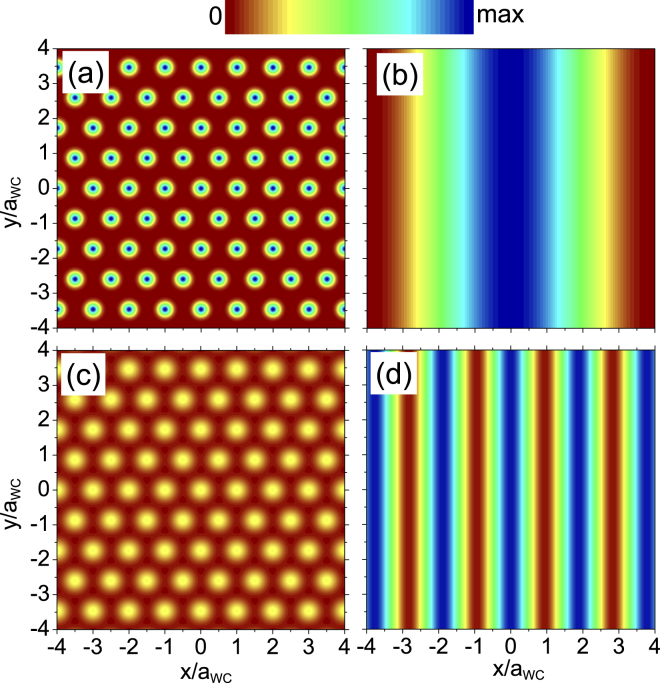
Electron density distribution *ρ*(***r***) at *r*_*s*_ = 60 for the (**a**,**c**) WC and (**b**,**d**) CDW phases near their mutual phase boundaries. Bilayer separation is (**a**,**b**) $d/{a}_{B}^{\ast }=5$ and (**c**,**d**) $d/{a}_{B}^{\ast }=10$. *a*_WC_ is the WC lattice constant.

Figure 3(a–d) show the CDW and c-WC electron bilayer density distributions at *r*_*s*_ = 60 for two values of the layer separation *d*, chosen close to the two CDW–c-WC phase boundaries. It is interesting to note how the periodicity of the CDW changes with *d*. The periodicity is longer for smaller *d*, and it evolves towards *a*_WC_ as *d* is increased. However even at the upper boundary of the CDW phase region at $d/{a}_{B}^{\ast }=10$, the value is still ${a}_{{\rm{CDW}}}\sim 2\,{a}_{{\rm{WC}}}$, (Fig. 3(d)). In Fig. 3(a,c), notice that the carriers in the WC become more localized on the lattice sites as *d* is decreased, due to the fact that for *r*_*s*_ = 60 the WC lies closer to the liquid phase boundary when $d/{a}_{B}^{\ast }=10$ than for $d/{a}_{B}^{\ast }=5$ (see Fig. 2).

**Figure 4:**
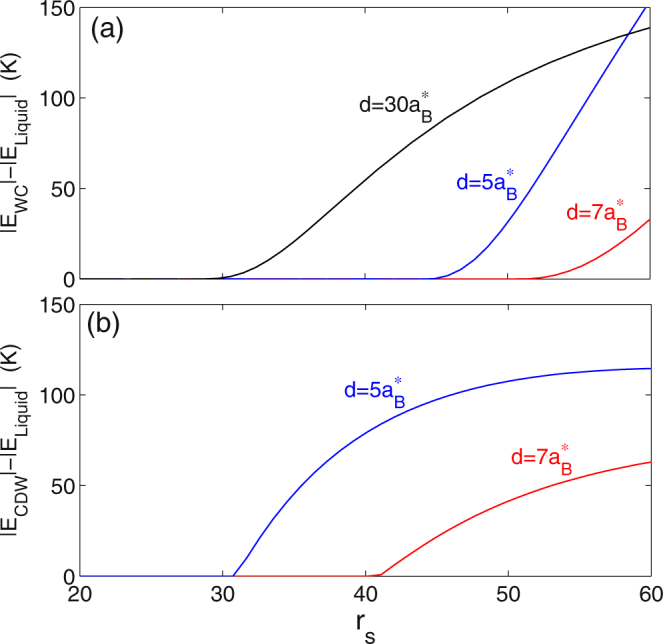
Energy difference between (**a**) the WC and Fermi liquid and (**b**) CDW and Fermi liquid, as function of *r*_*s*_ for different layer separations *d*, as labelled.

The differences in the total energies of the Fermi liquid, CDW, and c-WC phases provide us with an upper limit estimate of the transition temperatures. Figure 4(a,b) show the energy differences between the Fermi liquid and c-WC, and the Fermi liquid and CDW, respectively. Both sets of energy differences are consistently of the order of 50–100 K. This remains the case even for the largest layer spacings, $d/{a}_{B}^{\ast }\mathop{ > }\limits_{ \tilde {}}30$, where the c-WC approaches the uncoupled Wigner crystal. We thus expect that the transition temperatures for the CDW and c-WC phases to be comparable with the transition temperature for the uncoupled Wigner crystal.

## Discussion and Conclusions

The CDW phase could be identified using scanning tunneling microscopy (STM). The formation of the CDW along with the associated periodic lattice distortion opens a gap in the Fermi surface, modifying the local density-of-states, and this could be detected by tunneling^34–37^. A CDW phase can be also detected by transport measurements. 1D stripes may be pinned by disorder, in which case the CDW could be identified either with standard threshold voltage conductivity measurements or with frequency threshold measurements of ac conductivity^38^. A 1D-CDW breaks the rotational symmetry of the 2D plane, so it could manifest itself as a highly anisotropic transport response. Anisotropic resistivity as evidence of a CDW state has been experimentally studied, for example, in single crystals of *R*_2_CoGa_8_ (*R* = Gd, Tb, Dy, Ho, Er, Tm, Y, and Lu)^39^ and *R*Te_3_ (*R* = Y, La, Ce, Pr, Nd, Sm, Gd, Tb, Dy, Ho, Er, and Tm)^40^. However, there would not necessarily be any observable anisotropy, because the lack of an underlying anisotropic crystal structure means that no unique orientation of the 1D-CDW would be favored. Thus formation of randomly oriented domains of 1D-CDWs could occur. Further experimental evidence for the formation of a CDW state would be the observation of negative electronic compressibility in the bilayers. This can be measured via the difference between the actual differential capacitance and the classical geometric capacitance. The negative compressibility has been previously studied in double graphene monolayers in the presence of a perpendicular magnetic field^41^.

STM could also be used to observe a WC density profile. The c-WC phase could be also observed with transport measurements. Wigner crystallization should be accompanied by a transition to an insulating state, caused by pinning of the Wigner lattices by residual disorder^42, 43^. The c-WC phase can be distinguished from the CDW phase by observing differences in their low-lying excitation spectrum, since unlike the plasmon of the Fermi liquid phase, the low-energy collective mode for the WC is centred on a momentum transfer *q* equal to the reciprocal vector of the WC lattice^44^. This characteristic dispersion property of the collective mode should be readily observable in Raman spectra. Experimental investigation of optical phonon modes is an alternative approach to detect a c-WC phase as has been suggested for coupled 2D layers in semiconductor structures^45^.

Next we comment on the puzzling results reported from recent Coulomb drag experiments in coupled electron-hole graphene bilayers^46, 47^. A negative Coulomb drag was observed in two different temperature regimes, i.e. at low temperatures down to *T* = 1.5 K (ref. 46), and at high temperatures up to *T* = 170 K (ref. 47). Because the drag reported in ref. 47 was observed to be symmetric across the electron-hole and electron-electron systems, our inhomogeneous phases resulting from electron-hole correlations are unlikely to be the origin of the anomalous drag observed in coupled graphene bilayers.

In summary, we have proposed a system of two strongly coupled electron-hole bilayer graphene sheets as a promising candidate to observe new inhomogeneous c-WC and 1D-CDW phases which would interplay with the previously predicted electron-hole superfluid^23, 24^. We find in the strong interlayer coupling regime, that a 1D-CDW occurs at significantly higher densities than the c-WC phase.

There has been a long-standing issue in coupled electron-hole systems of whether a CDW phase would be two-dimensional or one-dimensional. A two-dimensional CDW phase would most likely retain the hexagonal structure of the 2D WC phase, but with a longer periodicity. We find that a hexagonal two-dimensional CDW phase has always a larger energy than both the Fermi liquid and 1D-CDW phases. We conclude from this that the hexagonal two-dimensional CDW phase would not be found as the ground state in coupled electron-hole layers.

## Electronic supplementary material

Supplementary Information
