# Supplementary material for: Inhomogeneous phases in coupled electron-hole bilayer graphene sheets: Charge Density Waves and Coupled Wigner Crystals
Source: Sci Rep. 2017 Sep 14;7:11510. doi: 10.1038/s41598-017-11910-w (PMC5599685; doi:10.1038/s41598-017-11910-w)
Supplement: Supplementary file 1 — Supplementary Information [file 41598_2017_11910_MOESM1_ESM.pdf]

Supplementary Information.
Inhomogeneous phases in coupled electron-hole bilayer graphene sheets: Charge Density Waves and Coupled Wigner Crystals

M. Zarenia, D. Neilson, F. M. Peeters

S1. CORRELATION ENERGY

An exact expression for the exchange-correlation functional $E_{xc}[\rho]$ can be written in terms of the coupling constant integral,[1, 2]

$$E_{\rm xc}[\rho] = \int_0^1 d\alpha\, W_\alpha[\rho]\ . \tag{S1}$$

$W_\alpha[\rho] = \langle \Psi^\alpha[\rho]|\hat{V}_{ee}|\Psi^\alpha[\rho]\rangle - E_{\rm coul}[\rho]$ is the potential energy functional, without the Hartree term, for a system with Coulomb-like interactions $\alpha\hat{V}_{ee} = \sum_{i<j}\alpha|\mathbf{r}_i - \mathbf{r}_j|^{-1}$ scaled by a multiplicative coupling constant α. The wave function Ψ^α minimizes the expectation value $\langle K[\rho] + \alpha\hat{V}_{ee}\rangle$ for this system, where K is the kinetic-energy operator and $\alpha\hat{V}_{ee}$ is the interaction. The ground-state density ρ is the same as for the real system where interactions are full-strength, i.e. $\alpha = 1$.

Requirements on the potential energy functional W_α are that it should be a smooth function of α, and that it should converge to $W_0 + W_0'\alpha$ in the limit $\alpha \to 0$ (weakly-interacting, high density limit) and to W_∞ in the opposite limit $\alpha \to \infty$ (strongly-interacting, low density limit). References [3–5] proposed the following interpolation,

$$W_\alpha[\rho] \simeq W_\infty + \frac{W_0 - W_\infty}{\sqrt{1+2X\alpha}}, \tag{S2}$$

where $W_0' = {\rm d}W_\alpha/{\rm d}\alpha|_{\alpha=0}$ and $X = W_0'/(W_\infty - W_0)$.

Substituting Eq. (S2) into Eq. (S1) gives for the correlation energy,

$$E_{\rm xc}[\rho] = W_0 + (W_0 - W_\infty)\left[\frac{\sqrt{1+2X}-1}{X} - 1\right]. \tag{S3}$$

To determine W_0 and W_0' we use the RPA for a coupled electron-hole BLG sheets given by Eqs. (2) and (3) in the manuscript. The RPA is exact in the limit of high-density.[7] Using RPA offers significant advantages over the approach of Ref. [4], which calculated W_0 for a 2DEG using the Fock integral with the occupied Kohn-Sham orbitals and which took W_0' as the second order coefficient in Görling-Levy perturbation theory [6], since our RPA calculations of W_0 and W_0' can readily be generalized to finite temperatures and to non-parabolic energy dispersions. To determine W_∞, we can represent the ground state of the classical WC for $\alpha \to \infty$ as an assembly of charged disks, as discussed in the manuscript.

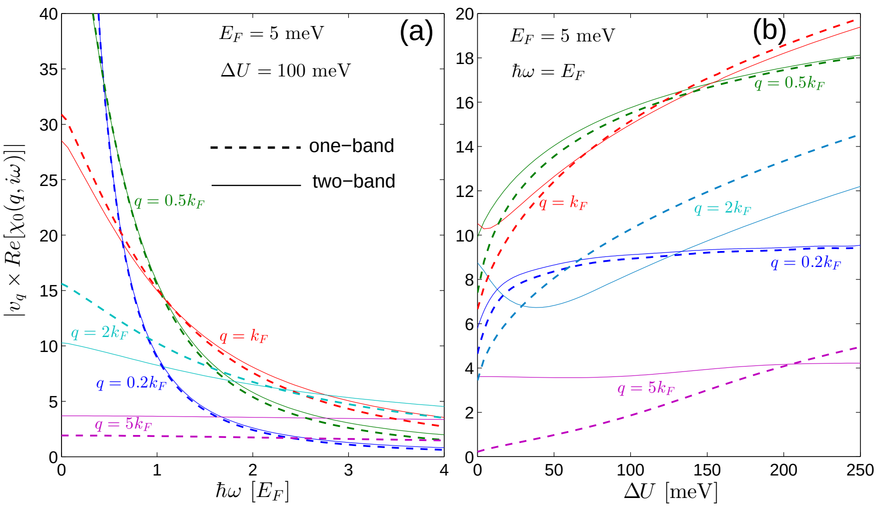

FIG. S1: Non-interacting density-density response function, Eq. (5) of the main text, as a function of (a) $\hbar\omega$ and (b) BLG gap ΔU for different values of q as labeled and with $E_F = 5$ meV, i.e. corresponding to the density $n \approx 1.5 \times 10^{11}$ cm^{-2}. The dashed curves are obtained by taking a parabolic band and $F_{\boldsymbol{k},\boldsymbol{k}+\boldsymbol{q}} = 1$. The solid curves are obtained by including both the electron and hole bands of gapped BLG (Eq. (6) of the main text).

S2. NON-INTERACTING DENSITY-DENSITY RESPONSE FUNCTION

In Fig. S1 we compare numerical results for the dynamical non-interacting density-density response function $\chi_0(q, i\omega)$ (Eq. (5) in the main text) when only one parabolic band is taken into account, with the corresponding results when the contributions of the electron and hole bands of the gapped BLG are both included (see Eq. (6) of the main text). For simplicity, here we have set the wave function overlap factor $F_{\boldsymbol{k},\boldsymbol{k}+\boldsymbol{q}}$ equal to unity. It can be seen, as expected, that the one-band model is a good approximation for $q \lesssim k_F$ when the gap in the BLG spectrum $\Delta U \gtrsim 50$ meV. We find that contributions to the integrals in Eqs. (2) and (3) of the main text become numerically unimportant when $q > k_F$. We conclude that we can neglect the influence of the hole band in the calculation of Eq. (5), provided the gap in the BLG spectrum is not too small.

[1] R. G. Parr and W. Yang, *Density-Functional Theory of Atoms and Molecules* (Oxford University Press, New York, 1989); R. M. Dreizler and E. K. U. Gross, *Density Functional Theory* (Springer, Berlin, 1990).
[2] P. Gori-Giorgi, M. Seidl, and G. Vignale, Phys. Rev. Lett. **103**, 166402 (2009).
[3] M. Seidl, Phys. Rev. A **60**, 4387 (1999).
[4] M. Seidl, J. P. Perdew, and M. Levy, Phys. Rev. A **59**, 51 (1999).
[5] M. Seidl and J. P. Perdew, Phys. Rev. B **50**, 5744 (1994).
[6] A. Görling and M. Levy, Phys. Rev. B **47**, 13105 (1993).
[7] G. Giuliani and G. Vignale, *Quantum Theory of the Electron Liquid* (Cambridge University Press, New York, 2008).
